# Optimization and Prediction of Ultimate Tensile Strength in Metal Active Gas Welding

**DOI:** 10.1155/2015/831912

**Published:** 2015-09-17

**Authors:** Anusit Ampaiboon, On-Uma Lasunon, Bopit Bubphachot

**Affiliations:** Manufacturing and Materials Research Unit, Faculty of Engineering, Mahasarakham University, Maha Sarakham 44150, Thailand

## Abstract

We investigated the effect of welding parameters on ultimate tensile strength of structural steel, ST37-2, welded by Metal Active Gas welding. A fractional factorial design was used for determining the significance of six parameters: wire feed rate, welding voltage, welding speed, travel angle, tip-to-work distance, and shielded gas flow rate. A regression model to predict ultimate tensile strength was developed. Finally, we verified optimization of the process parameters experimentally. We achieved an optimum tensile strength (558 MPa) and wire feed rate, 19 m/min, had the greatest effect, followed by tip-to-work distance, 7 mm, welding speed, 200 mm/min, welding voltage, 30 V, and travel angle, 60°. Shield gas flow rate, 10 L/min, was slightly better but had little effect in the 10–20 L/min range. Tests showed that our regression model was able to predict the ultimate tensile strength within 4%.

## 1. Introduction

Metal Active Gas (MAG) welding process, a subtype of Gas Metal Arc Welding (GMAW), has been used in welding industry for many decades due to its significant advantages, including high productivity, simple mechanism, good quality and mechanical properties of weld joint, and wide range of weldable materials and filler metals [1]. In MAG welding, a DC electric arc forms between a continuous filler electrode and a base metal. Heat is generated to fuse the metal in the joint area. Active shielding gas protects the molten weld pools and the electrode wire from contaminants in the atmosphere [1, 2].

In any welding process, welding parameters play important role in product quality as they affect mechanical properties and weld joint geometry [1–4]. However, selection of optimal parameters to meet the required specification is complicated as the weld quality can be affected by several variables, such as chemical compositions of workpiece material and wire electrode, shielding gas, and any heat treatment [1, 5]. Moreover, experimental optimization by trial and error is very time-consuming and costly [3, 4, 6]. Consequently, several methods and approaches such as Design of Experiment (DoE) and statistical techniques have been used to overcome this problem [1–3, 6]. Among the various methods used, a fractional factorial design has been widely used to identify significant process parameters and optimize product quality as it is useful for modelling and analyzing problems involving several parameters [7]. Several research studies have focused on optimizing welded bead geometry and weld joint mechanical properties [1–6, 8].

In this work, the fractional factorial design was used to determine the effect of MAG welding parameters on ultimate tensile strength (UTS) of mild steel. Tensile strength was selected to assess weld quality because it is a key mechanical property that can describe weld joint performance [6]. The UTS of weld joint is important because it is an estimate of the maximum load that the weld can support [5]. The optimal parameters for maximum UTS were also considered. Analysis of variance (ANOVA) and regression analysis were used to determine the significant parameters and to develop a model for the UTS.

## 2. Experimental Details

### 2.1. Specimen Preparation and Testing

**Figure 1:**
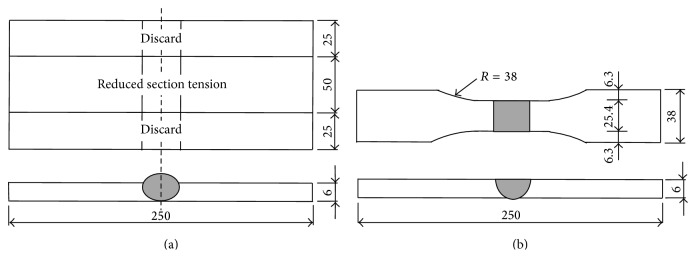
Schematic diagram of (a) zero-gap butt joint welding and (b) tensile strength test sample (unit: mm).

Mild steel (ST37-2) 6 mm thick was the base metal; it has yield strength of 340 MPa and ultimate tensile strength of 470 MPa: its chemical composition is shown in Table 1. 125 mm × 100 mm specimens were prepared and cleaned with a brush for better weld quality. A zero-gap butt joint was formed in a single pass by MAG welding; see Figure 1(a). After welding, samples for ultimate tensile strength (UTS) test were cut in accordance with the ASME IX standard; see Figure 1(b). The UTS was tested at room temperature using a 10-ton capability universal testing machine.

### 2.2. Equipment

A WIM welding machine MIG 350SEF, with constant voltage power source and direct current electrode positive (DCEP) polarity was used for welding. An electrode wire, AWS A5.18 ER70S-6, with 0.8 mm rod diameter was the welding consumable. Chemical composition of this electrode is shown in Table 1. Commercial carbon dioxide (99.98% CO_2_) was used as the shielding gas to prevent chemical reactions between the hot workpiece surface and the atmosphere.

### 2.3. Selection of Welding Parameters and Their Levels

Six input parameters were investigated with feasible ranges recommended by a welding handbook [9] and limited by the machine capabilities. The two levels of the input factors used are shown in Table 2.

### 2.4. Experimental Design

The experiment used a 2^6−2^ fractional factorial design with 16 combinations. Two replicates were run for each combination, giving a total of 2 × 16 = 32 experiments. The experimental layout was generated by MINITAB software [10] where systematic error was avoided by random parameter assignment; see Table 3.

## 3. Result and Discussion

### 3.1. Experimental Result

Table 3 shows our results. The statistical software, MINITAB, analyzed the data and generated the model for the UTS.

### 3.2. Analysis of Variance for UTS

**Figure 2:**
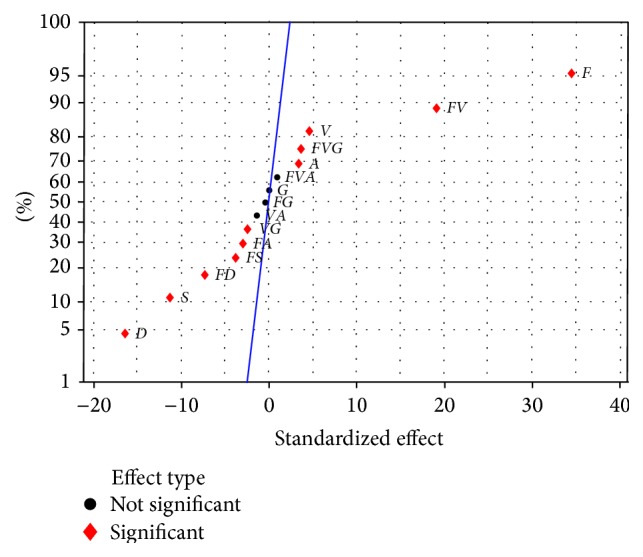
Normal probability plot of the effects (at *α* = 0.05).

A normal probability plot of the effects in Figure 2 was used to visually identify important effects on the UTS. Important effects are large and further from the fitted line while unimportant effects are smaller and centered around zero [7, 10]. In Figure 2, the significant effects that emerge from this analysis are the strong effect of *F*, *V*, *S*, *A* and *D*; the 2-way interactions, *FD*, *FS*, *FA*, *FV*, and *VG*, and the 3-way interaction, *FVG*. The insignificant effects are the weak effect of *G* and the interactions *FG*, *VA*, and *FVA*. All of the insignificant effects should be removed from the analysis, but weak effects should not be removed when they are involved in significant interactions [8]. Therefore, the weak effect *G* is included in the model due to its significant interactions (*VG* and *FVG*).

After the insignificant terms were removed, the significance of the reduced UTS model was tested; see Table 4. The estimated effects and the coefficients of the reduced model are also given in Table 4. A higher absolute value of the estimated effect indicates a greater influence of that model term on the UTS. Consequently, it was evident that wire feed rate (*F*) showed the greatest effect on the UTS of weld joint, followed by interaction of wire feed rate and welding voltage (*FV*), tip-to-work distance (*D*), welding speed (*S*), interaction of wire feed rate and tip-to-work distance (*FD*), welding voltage (*V*), interaction of wire feed rate and welding speed (*FS*), 3-way interaction of wire feed rate-welding voltage and shielding gas flow rate (*FVG*), travel angle (*A*), interaction of wire feed rate and travel angle (*FA*), interaction of welding voltage and gas flow rate (*VG*), and shielding gas flow rate (*G*), respectively. This conclusion was graphically presented in Figure 2. The extremely low *p* value, much less than 0.05, implied that the model term was highly significant. The coefficient of determination, *R* ^2^, of 0.9907 was in reasonable agreement with the adjusted *R* ^2^ of 0.9848. Therefore, the reduced model terms appeared to be statistically adequate to develop the prediction model for the UTS.

### 3.3. Model for UTS

Multiple regression analysis for the prediction of UTS was conducted on the experimental data in Table 3. The regression model (uncoded units) in (1) was developed by calculating regression coefficients of the reduced model terms:

(1) UTSun-coded unit=288.912−6.79774F−17.5458V−0.0681944S+2.90104A−0.62240D+7.97500G+1.92756F∗V−0.0122222F∗S−0.138542F∗A−0.908854F∗D−0.325V∗G+0.00038462F∗V∗G,

where UTS is ultimate tensile strength (MPa), *F* is wire feed rate (m/min), *V* is welding voltage (volt), *S* is welding speed (mm/min), *A* is travel angle (degree), *D* is tip-to-work distance (mm), and *G* is shielded gas flow rate (liter/min).

In running a two-level factorial experiment, we usually fit a first-order model which includes only the main effects and interaction terms [7]; see (1).

### 3.4. UTS Optimization of Welding Parameters

**Figure 3:**
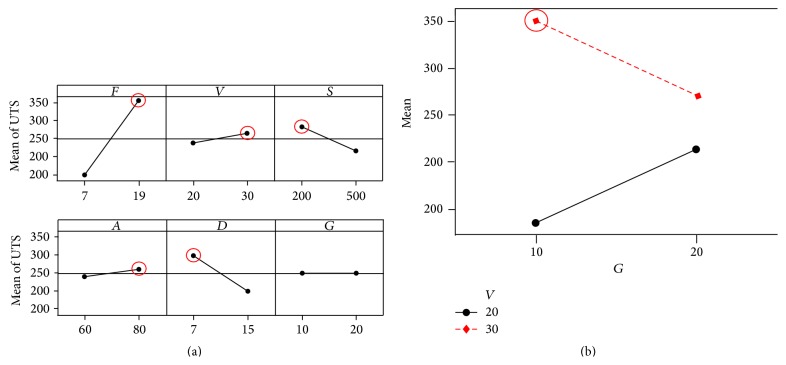
(a) Main effects plot and (b) interaction plot of welding voltage and gas flow rate (*VG*) for UTS.

The maximum UTS was the single objective of this study. To select the optimal welding parameters, the main effect of each parameter in Figure 3(a) was considered. Figure 3(a) revealed that wire feed rate (*F*), welding voltage (*V*), and travel angle (*A*) had positive effects on the UTS and increasing these variables leads to larger UTS. In contrast, welding speed (*S*) and tip-to-work distance (*D*) had negative effects—increasing these variables reduced the UTS. Shield gas flow rate (*G*) within the 10–20 L/min range showed less effect; thus the significant VG interaction plot was used to determine the optimum condition.  Figure 3(b) showed that welding voltage had a large effect at low gas flow rate, but smaller effect at high gas flow rate. As the maximum UTS was desired, the optimum process parameters for MAG welding were wire feed rate at 19 m/min, welding voltage at 30 volts, welding speed at 200 mm/min, travel angle at 60 degrees, tip-to-work distance at 7 mm, and shielded gas flow rate at 10 liters/min; see Figure 3. The maximum UTS calculated from (1) was 553 MPa.

### 3.5. Confirmation Test

To verify the multiple regression model in (1) and the optimization of welding parameters, six experiments used the optimal welding conditions. UTS obtained by the predicted model and the experiments were compared and percentage errors are shown in Table 5. The predicted UTS agreed well with the measured UTS. Deviations were between −2% and 4%.

Noticeably, the maximum UTS obtained from the experiment (558.3 MPa on average) was larger than the UTS of the base metal (470 MPa). During tensile test, it was observed that tearing of welded specimen occurred at the weld joint rather than at the base metal. It was important to point out that high UTS could be obtained even when bead penetration was not completely full through the entire thickness of the workpiece.

## 4. Conclusions

We applied a fractional factorial design to zero-gap butt welding of mild steel using MAG. The study focused on the effect of welding parameters on the ultimate tensile strength (UTS) of the welded joint and the optimal welding conditions for maximum UTS. We conclude the following:

1. Process parameters that showed the greatest to the least effects on UTS of welded joint were in the order of welding feed rate, tip-to-work distance, welding speed, welding voltage, and travel angle. Shield gas flow rate in the selected range was found to have little effect.
2. UTS increased with increased welding feed rate, welding voltage, and travel angle. In contrast, UTS increased with decreased welding speed and tip-to-work distance.
3. The maximum UTS of a welded joint, 558 MPa on average, was obtained at wire feed rate = 19 m/min welding voltage = 30 V, welding speed = 200 mm/min, travel angle = 60°, tip-to-work distance = 7 mm, and shield gas flow rate = 10 L/min.
4. Maximum UTS results from the regression model agreed with experiments within 4%. Therefore, this model may be used to predict weld UTS with sufficient accuracy.

It is important to point out that the model obtained from this investigation is the first-order model in which only the main effect and the interaction terms are included. However, there is a possibility that the second-order model or a nonlinear model is more appropriate. Therefore, we plan to study response surface methods (RSM) to investigate process optimization.

## Figures and Tables

**Table 1:** Chemical compositions of base metal and electrode wire (% weight).

Material	S	Si	Mn	C	P	Fe
ST37-2 base metal	0.003	0.04	0.82	0.12	0.012	Balance
ER70S-6 electrode	0.01	0.58	1.15	0.08	0.014	Balance

**Table 2:** Welding input parameters and their levels.

Number	Parameter	Unit	Notation	Low level (−1)	High level (+1)
1	Wire feed rate	m/min	*F*	7	19
2	Welding voltage	Volt	*V*	20	30
3	Welding speed	mm/min	*S*	200	500
4	Travel angle	Degree	*A*	60	80
5	Tip-to-work distance	mm	*D*	7	15
6	Shield gas flow rate	Liter/min	*G*	10	20

**Table 3:** Experimental design matrix and results.

Run order	*F*	*V*	*S*	*A*	*D*	*G*	UTS [MPa]	Run order	*F*	*V*	*S*	*A*	*D*	*G*	UTS [MPa]
1	1	−1	1	−1	−1	1	173	17	1	1	−1	1	−1	−1	211
2	1	1	−1	−1	−1	1	265	18	1	−1	−1	1	1	1	250
3	−1	−1	−1	−1	−1	−1	57	19	−1	−1	1	−1	1	1	73
4	1	−1	1	1	−1	−1	533	20	−1	−1	−1	−1	−1	−1	549
5	−1	1	1	1	−1	1	124	21	1	1	−1	1	−1	−1	137
6	1	1	1	1	1	1	278	22	−1	1	−1	−1	1	1	325
7	−1	1	−1	−1	1	1	124	23	−1	−1	1	1	1	−1	93
8	1	−1	1	1	−1	−1	306	24	−1	−1	1	−1	1	1	306
9	1	−1	−1	−1	1	−1	307	25	−1	1	1	−1	−1	−1	264
10	1	−1	−1	−1	1	−1	246	26	1	−1	−1	1	1	1	263
11	1	1	1	−1	1	−1	122	27	−1	−1	−1	1	−1	1	124
12	1	1	1	1	1	1	551	28	−1	1	−1	1	1	−1	537
13	−1	−1	1	1	1	−1	151	29	−1	1	1	−1	−1	−1	135
14	−1	1	1	1	−1	1	312	30	1	1	1	−1	1	−1	321
15	−1	1	−1	1	1	−1	118	31	1	1	−1	−1	−1	1	80
16	1	−1	1	−1	−1	1	318	32	−1	−1	−1	1	−1	1	306

**Table 4:** Estimated effects and coefficients for UTS (coded units).

Term	Effect	Coefficient	*t*-statistic	*p* value
Constant		248.69	80.45	<0.001
Wire feed rate, *F*	210.751	105.38	34.09	<0.001
Welding voltage, *V*	27.12	13.56	4.39	<0.001
Welding speed, *S*	−68.13	−34.06	−11.02	<0.001
Travel angle, *A*	22.00	11.00	3.56	0.002
Tip-to-work distance, *D*	−99.50	−49.75	−16.09	<0.001
Shield gas flow rate, *G*	−0.25	−0.13	−0.04	0.968
*FV*	116.00	58.00	18.76	<0.001
*FS*	−22.00	−11.00	−3.56	0.002
*FA*	−16.63	−8.31	−2.69	0.015
*FD*	−43.63	−21.81	−7.06	<0.001
*VG*	−16.00	−8.00	−2.59	0.018
*FVG*	21.12	10.56	3.42	0.003

*S* = 17.4857 *R* ^2^ = 99.07%  *R* ^2^ (adj.) = 98.48%, coefficient standard error = 3.1.

**Table 5:** Results of confirmation experiment for UTS.

Experiment number	Number 1	Number 2	Number 3	Number 4	Number 5	Number 6	Mean	SD
Actual value [MPa]	541	537	564	564	569	575	558.3	15.6
Predicted value [MPa]	553	553	553	553	553	553		
% Error^*∗*^	−2	−3	2	2	3	4		

^*∗*^Percentage error was calculated as %Error = ((Actual  value − Predicted  value)*∗*100)/Predicted  value.
